# Antimicrobial stewardship knowledge, attitudes, and practices (KAP) among nurses

**DOI:** 10.1017/ash.2024.63

**Published:** 2024-04-18

**Authors:** Reinaldo Perez, Jillian E. Hayes, Ali R. Winters, Rebekah H. Wrenn, Rebekah W. Moehring

**Affiliations:** 1 Division of Infectious Diseases, Department of Medicine, Duke University Medical Center, Durham, NC, USA; 2 Duke Center for Antimicrobial Stewardship and Infection Prevention, Durham, NC, USA; 3 Department of Pharmacy, Duke University Medical Center, Durham, NC, USA; 4 Department of Nursing, Duke University Medical Center, Durham, NC, USA

## Abstract

We performed a knowledge, attitudes, and practice (KAP) survey of bedside nurses to evaluate perceptions of antimicrobial use and aid in the design of nursing-based antimicrobial stewardship interventions. The survey highlighted discrepancies in knowledge and practice as well as opportunities to improve communication with nursing colleagues.

## Introduction

In 2017, the Centers for Disease Control and Prevention (CDC) and the American Nurses Association (ANA) published a white paper emphasizing the opportunity to redefine the antimicrobial stewardship program (ASP) by including bedside nurses in stewardship initiatives.^1,2^ This multidisciplinary approach to stewardship is now considered essential—both the CDC and the Joint Commission standards list nursing services as key members of the stewardship team.^3^ The ANA identified several potential stewardship functions for bedside nurses, but the feasibility and efficacy of these proposed interventions vary depending on the local environment.^4^

Previous Knowledge, Attitudes, and Practice (KAP) surveys have investigated nursing familiarity with antibiotic stewardship. These investigations demonstrated nurses appreciated the importance of antimicrobial resistance (AR) and inappropriate antibiotic prescribing, but were unfamiliar with ASPs and desired further education on antibiotics.^5–7^ We aimed to build on this work and use a nursing KAP survey to guide stewardship implementation.

Based upon CDC/ANA recommendations^1^ and facility-specific needs, we identified four potential topics amenable to a nursing-based intervention: administration of beta-lactams before vancomycin in patients presenting with sepsis, prompting intravenous (IV) to oral (PO) transitions, diagnostic stewardship of urine culture collection, and penicillin allergy assessment.^2^ We sought to characterize the KAP of our nursing staff pertaining to these four stewardship topics to inform future stewardship interventions.

## Methods

Given the limited availability of validated tools we created a novel, 24-question KAP survey assessing overall familiarity with antimicrobial stewardship in four target content areas: antibiotic infusion sequence, IV to PO transitions, indications for urine culture, and penicillin allergy assessment The voluntary survey invitation was distributed by email listservs from key nursing leaders to approximately 2,900 employed bedside nurses at Duke University Hospital, a 1,000-bed academic center in Durham, North Carolina. Responses were collected digitally with distribution using the Qualtrics platform over the period of January to March of 2023. Descriptive statistics were calculated using the integrated Qualtrics analytics platform. The survey and data collection processes were approved by the Duke University Institutional Review Board. Full methods and a copy of the survey are available in the attached supplement.

## Results

We received a total of 85 completed survey responses, a response rate of 2.9%. Respondents were primarily dayshift (n = 59, 69%), staff nurses (n = 56, 66%), from med/surg floors (n = 68, 80%). Respondents had a median 9 years of experience in nursing (IQR 3–17) and most had a highest level of education of at least bachelor’s degree (n = 77, 91%). Full demographic details are available in the supplement.

The majority (87%) of survey respondents agreed they had responsibility to contribute to appropriate antimicrobial use and 92% reported that having strong knowledge of antimicrobials was important to their role. However, 85% of nurses indicated that they would like more education on the appropriate use of antimicrobials.

Nurses were generally familiar with the challenges posed by AR: 92% of respondents agreed AR is a significant problem nationally and 86% of participants agreed that ASPs improve patient care. However, when applying these principles to the local environment of their own hospital, nurses perceived less of a threat; 35% of participants agreed that AR is a significant problem at their institution (Table 1). A similar discrepancy was seen in the perceived harms of antimicrobial use. Participants were generally unfamiliar with ASP resources with only 6% of respondents reporting familiarity with the internal ASP website housing facility guidelines and dosing recommendations, 4% being familiar with the facility antibiogram, and 6% reporting familiarity with the ASP pager.

**Table 1.** Nursing perception of antimicrobial resistance and stewardship nationwide vs local institution

Statement	Nationally agree or strongly agree, n (%)	At your hospital agree or strongly agree, n (%)
Antimicrobial resistance is a significant problem	78 (92)	30 (35)
Antimicrobial use can harm patients	59 (69)	22 (26)
Antimicrobials are overused	58 (68)	52 (61)
Selecting broad-spectrum antimicrobials when narrower spectrum antimicrobials are available contributes to antimicrobial resistance	68 (80)	57 (67)
Antimicrobial resistance can be reduced by improving infection control practices	79 (93)	78 (92)
Development of new antimicrobials will keep up with increasing resistance	43 (51)	---
Antimicrobial stewardship programs improve patient care and safety	73 (86)	71 (84)

Note. *N* = 85 total respondents, Duke University Hospital.

**Figure 1.**
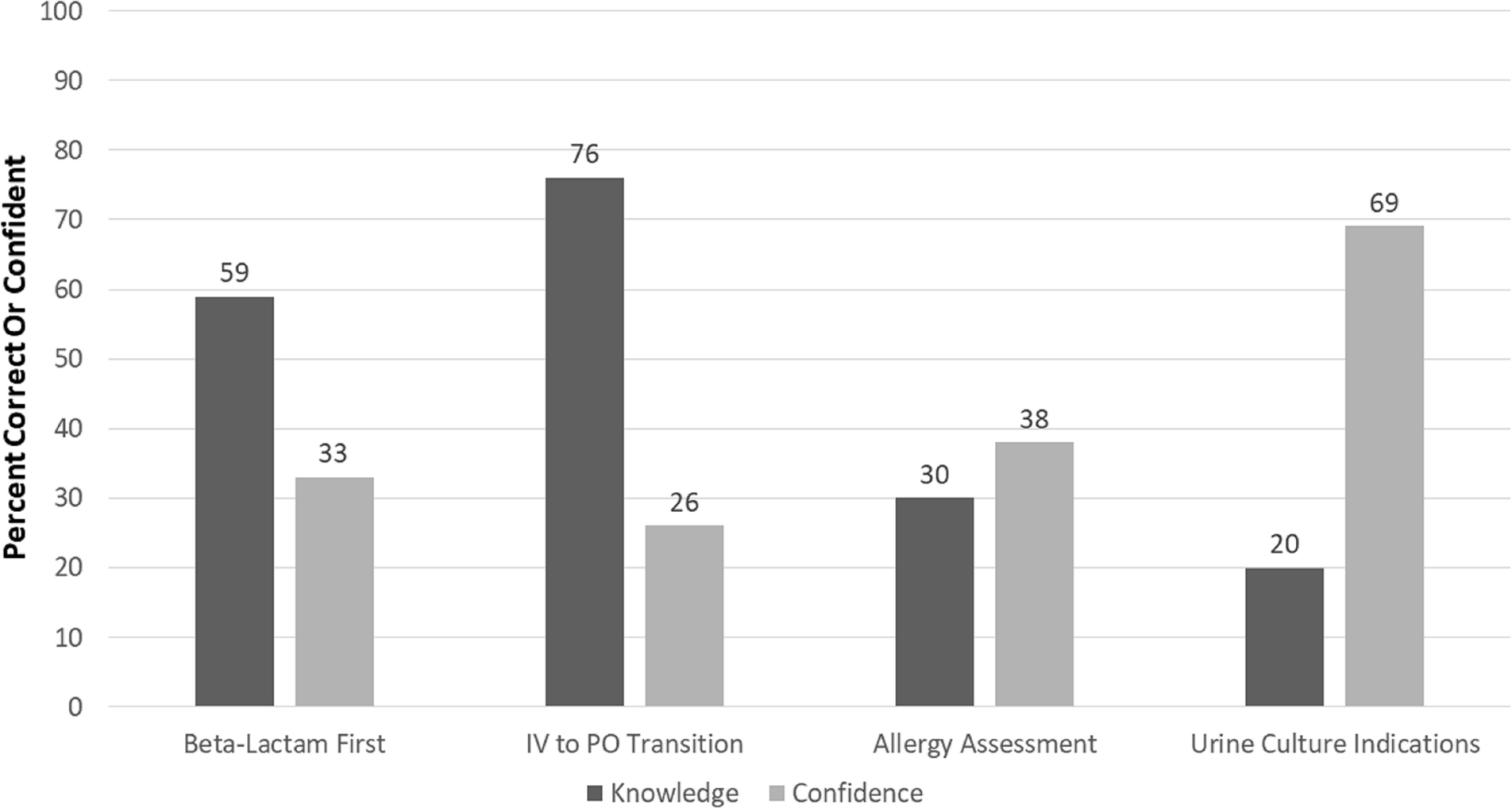
Knowledge vs confidence in antimicrobial stewardship topic areas. *Note:* N = 85 total respondents, Duke University Hospital. Beta Lactam First refers to nursing knowledge of and confidence in order of antimicrobial infusion in sepsis. IV to PO Transition refers to nursing knowledge and confidence in antimicrobials which can safely be transitioned from IV to PO with similar efficacy. Allergy Assessment refers to nursing knowledge of and confidence in penicillin allergy delabeling. Urine Culture Indications refers to nursing knowledge of and confidence in appropriate indications for urine culture.

Respondents generally lacked confidence in decision-making required for the four topic areas identified in the survey: 26% reported confidence in identifying IV to PO switches, 38% reported comfort with allergy assessments, 33% were confident with beta lactam administration first decisions. Highest confidence (69%) was reported in identifying appropriate indications for urinalysis and culture. Participant performance on knowledge-based questions in these four topic areas frequently contrasted with the relative level of confidence (Figure 1), particularly for urine culture indications where 20% of respondents answered knowledge questions incorrectly despite reported high confidence in this domain.

## Discussion

This nursing KAP survey highlighted the discrepancy between nursing confidence versus demonstrable knowledge in clinical decision-making relevant to four potential antimicrobial stewardship initiative topic areas. Further, differences were seen in the national versus local perceptions of antimicrobial resistance and the threat it may pose. Additionally, we found that nursing staff were largely unfamiliar with the suite of ASP resources available at our facility. We found these results deeply informative to the design of future stewardship interventions targeted at bedside nurses who serve as our most important patient advocates and educators.

Previous KAP surveys of nurses describe nurses’ overall familiarity with antimicrobial stewardship, attitudes toward AR on the national level, and understanding of antimicrobials.^5–7^ Our study builds on this experience and demonstrates results can change substantially when framed on the local level. We designed our study with specific ideas for future nurse-driven stewardship interventions in mind, allowing us to shed light on potential challenges and opportunities to their implementation. Finally, while other studies have asked about general educational resources our survey highlighted the challenges of disseminating ASP educational materials to bedside nurses.

For our ASP, the finding of greatest relevance was the discrepancy between nursing confidence and knowledge in our four topic areas for proposed interventions. For a nursing-based IV to PO initiative, our data suggested we focus on empowering nurses to feel confident in their assessments and in prompting a switch to oral medications. In contrast, for a urine culture diagnostic stewardship intervention our data suggested a need to focus on education to overcome misconceptions about indications for urinalysis and culture. Further, this educational initiative would need to be communicated in a novel manner given nursing unfamiliarity with our existing ASP resources. Nursing-based stewardship interventions proposed at the national level are better informed by adaptation to local environments. For this purpose, KAP surveys remain a helpful method to anticipate implementation challenges in a specific setting. We believe ASP personnel aiming to engage multidisciplinary groups in stewardship should prioritize gathering KAP data when designing inclusive initiatives.

Our study has limitations. The survey was conducted at a single large academic center with a well-supported ASP and magnet level nursing staff; thus, results may not be reflected in other, more diverse institutions such as of smaller community hospitals. Additionally, our knowledge assessment for stewardship topics was not a previously validated tool and may be an imperfect measure of clinical decision-making in real-world scenarios. Finally, the survey was voluntary with a low response rate and may not be representative of nurses as a whole with insufficient power to evaluate differences which could exist in nurses from distinct work areas.

Nearly all patient care flows through bedside nurses, who represent critical partners and a valuable resource to expand the reach of antibiotic stewardship initiatives and promote the principles of judicious antibiotic use. The success of these multidisciplinary interventions will depend on improving ASP communication with bedside nurses and an understanding of stewardship knowledge, attitudes and practices at the local level. ASPs should consider the use of KAP surveys to guide their development of nursing-based stewardship initiatives.

## Supporting information

Perez et al. supplementary material Perez et al. supplementary material

## Supplementary material

For supplementary material accompanying this paper visit http://doi.org/10.1017/ash.2024.63. click here to view supplementary material
