# Supplementary material for: Antimicrobial stewardship knowledge, attitudes, and practices (KAP) among nurses
Source: Antimicrob Steward Healthc Epidemiol. 2024 Apr 18;4(1):e51. doi: 10.1017/ash.2024.63 (PMC11036424; doi:10.1017/ash.2024.63)
Supplement: Perez et al. supplementary material [file S2732494X24000639sup001.docx]

**Supplementary Methods Document**

**Antimicrobial Stewardship Knowledge, Attitudes, and Practices (KAP) Among Nurses**

**Design:** Target population was nurses at Duke University Hospital working in adult units. This included med-surg units, critical care units and the emergency department.

**IRB approval and informed consent process:** The study was reviewed by the Duke IRB and deemed to be exempt. Study was voluntary and a consent statement was present at the beginning of the survey (see copy of survey below). No personal information was collected and data was restricted to the study team.

**Development and pre-testing:** A literature search was performed to look for examples of nursing KAP surveys focused on antimicrobial stewardship. Using these as templates we adapted the instrument to better focus on the potential areas for intervention suggested by the CDC/ANA white paper. The CDC/ANA list is extensive, and to aid in creation of a less burdensome survey the stewardship team chose the four topic areas felt most feasible to design a nursing-based stewardship intervention at our facility. These areas were: 1) nursing identification of antimicrobials appropriate for IV to PO transition and prompting switch, 2) nursing adherence to infusion of beta lactams first in the setting of sepsis, 3) a nursing driven diagnostic stewardship intervention to reduce inappropriate urine cultures, and 4) nursing based evaluations of penicillin allergies and referral for penicillin allergy de-labeling. Drafts of the survey were piloted by nursing members of our stewardship team, on groups of floor nurses, and nursing leadership in an iterative process until all parties were satisfied with the end product. The final survey was approved by numerous hospital committees.

**Recruitment process:** The survey was open to all who had the unique survey link. Contact was made using hospital nursing mailing lists. The survey was advertised at morning huddles and routine nursing conferences as well as with a series of emails.

**Survey Administration:** The survey was distributed via email and administered using the Qualtrics web platform. The survey was voluntary but incentivized with the potential to earn a meal voucher for use at the hospital cafeteria. Reminders about the survey were distributed by high level nursing leadership including the chief nursing officer. The survey was administered from January to March of 2023. There was no randomization of the items or use of adaptive questioning in our survey. The survey consisted of 24 items over 4 pages. Only surveys which were fully completed were included in the analysis. Participants were given a review option.

**Response Rates:** Our email-based survey platform did not allow for quantification of a view rate or a participation rate.

**Preventing Multiple entries:** To receive the incentive meal voucher participants entered their Duke university email address at the end of the survey and this was monitored for duplicates. More advanced techniques such as cookies or IP tracking were not utilized. Given the email-based distribution we felt false entries were low risk.

**Analysis:** Only completed surveys were included. Given the shift work nature of nursing there was not exclusion of atypical timestamps. No statistical correction was utilized.

**Our ASP:** The Duke University Hospital Antimicrobial Stewardship Evaluation Team is supported by 1.25 adult and pediatric infectious disease (ID) faculty physicians, 0.5 ID fellows, and 3.0 ID pharmacists. The group reports to the hospital pharmacy and therapeutics committee and provides a wide array of stewardship support including clinical practice guidelines, development of clinical decision support and handshake rounds in the hospital ICUs. A unique part of the program is a dedicated consult pager to talk with an infectious diseases pharmacist for brief questions about drug selection or dosing which do not require full infectious diseases consultation.

**Copy of Distributed Survey:**

**Antimicrobial Stewardship Knowledge, Attitudes, and Practices (KAP) Survey**

Hello!

You are being asked to participate in a research study, which focuses on designing sustainable, nursing-driven antimicrobial stewardship interventions at your institution. By completing this survey, you are consenting to participate in this study. This study is completely voluntary and there are no potential risks to participation. We are interested in providing the best possible antimicrobial stewardship education to nurses while best understanding and respecting your current workflow.

Your answers to this survey will be recorded anonymously and will be used to guide the development of educational tools and future antimicrobial stewardship interventions at Duke University Hospital. It will take you approximately 10-15 minutes to complete this survey.

Thank you for participate in this survey and sharing your perspective with us!

Duke Antimicrobial Stewardship and Education Team (ASET)

***Demographic Information***

Please answer the following demographic questions to the best of your ability. As a reminder, all responses are recorded anonymously.

1. Please select the classification that best represents the unit you work on most frequently:
   1. Medicine/Pulmonary
   2. Surgery (including OR, PACU)
   3. Oncology
   4. Critical Care
   5. Cardiology
   6. Neurology
   7. Emergency Medicine
   8. OB/GYN
   9. Anesthesiology
   10. Administration
   11. Infection Prevention
   12. Transplant
   13. Other (please describe):
2. During which shift do you work the majority of your hours?
   1. First
   2. Second
   3. Third
3. Please indicate your highest degree:
   1. Diploma
   2. Associates
   3. Bachelors
   4. Masters or higher
   5. Licensed practical nurse (LPN)
4. Please select your primary role (in which you work the majority of your shifts in a 30-day time period):
   1. Staff nurse
   2. Preceptor
   3. Charge nurse
   4. Nurse manager
   5. Infection prevention
   6. Traveler/contractor
5. How many years have you practiced nursing (post-completion of the degree listed in question 3)?
   1. <1 year
   2. 1-3 years
   3. 4-5 years
   4. 5-10 years
   5. 10-19 years
   6. >19 years
6. How many years have you practiced nursing at **this** institution?
   1. < 5 years
   2. 5-10 years
   3. 10-19 years
   4. >19 years

The following statements reflect your opinions related to current medical practice in the United States.

| **Statement** | **Strongly Agree** | **Agree** | **Neutral** | **Disagree** | **Strongly Disagree** |
| --- | --- | --- | --- | --- | --- |
| Antimicrobial resistance is a significant problem nationally. | 1 | 2 | 3 | 4 | 5 |
| Antimicrobial use can harm patients. | 1 | 2 | 3 | 4 | 5 |
| Antimicrobials are overused nationally. | 1 | 2 | 3 | 4 | 5 |
| Selecting broad-spectrum antimicrobials when narrower spectrum antimicrobials are available contributes to antimicrobial resistance. | 1 | 2 | 3 | 4 | 5 |
| Antimicrobial resistance can be reduced by improving infection control practices. | 1 | 2 | 3 | 4 | 5 |
| Development of new antimicrobials will keep up with increasing resistance. | 1 | 2 | 3 | 4 | 5 |
| Antimicrobial stewardship programs reduce the problem of antimicrobial resistance. | 1 | 2 | 3 | 4 | 5 |
| In general, antimicrobial stewardship programs improve patient care and safety. | 1 | 2 | 3 | 4 | 5 |

The following statements are about your hospital in general.

| **Statement** | **Strongly Agree** | **Agree** | **Neutral** | **Disagree** | **Strongly Disagree** |
| --- | --- | --- | --- | --- | --- |
| Antimicrobial resistance is a significant problem at my institution. | 1 | 2 | 3 | 4 | 5 |
| Antimicrobials are overused at my institution. | 1 | 2 | 3 | 4 | 5 |
| Antimicrobial use can harm patients at my institution. | 1 | 2 | 3 | 4 | 5 |
| Selecting broad-spectrum antimicrobials when narrower spectrum antimicrobials are available contributes to antimicrobial resistance at my institution. | 1 | 2 | 3 | 4 | 5 |
| Antimicrobial resistance can be reduced by improving infection control practices at my institution. | 1 | 2 | 3 | 4 | 5 |
| In general, the antimicrobial stewardship program at my institution improves patient care and safety. | 1 | 2 | 3 | 4 | 5 |

The following statements are about your personal experiences at your hospital.

| **Statement** | **Strongly Agree** | **Agree** | **Neutral** | **Disagree** | **Strongly Disagree** |
| --- | --- | --- | --- | --- | --- |
| When giving a patient an antimicrobial, I know why the patient is receiving the medication. | 1 | 2 | 3 | 4 | 5 |
| It is my responsibility to contribute to appropriate antimicrobial use at my hospital. | 1 | 2 | 3 | 4 | 5 |
| I would like more education on the appropriate use of antimicrobials. | 1 | 2 | 3 | 4 | 5 |
| Strong knowledge of antimicrobials is important in my role. | 1 | 2 | 3 | 4 | 5 |

The following statements seek to assess your confidence with different aspects of antimicrobials. The results will be used to develop future educational tools.

| **Statement** | **Strongly Agree** | **Agree** | **Neutral** | **Disagree** | **Strongly Disagree** |
| --- | --- | --- | --- | --- | --- |
| I am confident that I know when to recommend conversion of intravenous antibiotics to an oral formulation. | 1 | 2 | 3 | 4 | 5 |
| I am confident that I know how to perform a thorough antimicrobial allergy assessment. | 1 | 2 | 3 | 4 | 5 |
| I am confident that I know the best order to administer medications when vancomycin and a beta-lactam are ordered to start at the same time in a patient presenting with infection. | 1 | 2 | 3 | 4 | 5 |
| I am confident that I know when to obtain a urinalysis for a patient. | 1 | 2 | 3 | 4 | 5 |
| I am confident that I can categorize antimicrobials appropriately (e.g., beta-lactams, fluoroquinolones, etc.) | 1 | 2 | 3 | 4 | 5 |

The following statements/questions seek to assess your knowledge and practices related to antimicrobials. The results will be used to develop future educational tools.

1. Which of the following antibiotics is a beta-lactam?
   1. Azithromycin
   2. Doxycycline
   3. Levofloxacin
   4. **Piperacillin/tazobactam**
2. Which of the following antibiotics can readily be converted from IV to oral at a 1:1 dosing conversion?
   1. **Azithromycin**
   2. Cefepime
   3. Meropenem
   4. Piperacillin/tazobactam
3. Select the patient below who represents the most ideal patient to consider conversion from IV to oral antimicrobials.
   1. 79-year-old male with bloodstream infection caused by carbapenem-resistant *Acinetobacter baumanii* with most recent blood pressure of 78/40
   2. **40-year-old male with community-acquired pneumonia, clinically stable, nearing discharge, tolerating multiple other oral medications**
   3. 55-year-old female with an intra-abdominal abscess, awaiting a trip to the operating room for source control, inability to keep any fluids down
   4. 65-year-old female with ventilator-associated pneumonia, currently without oral access, admitted to the medical intensive care unit (MICU)
4. You are caring for a patient presenting with sepsis from a urinary source. The primary team has ordered both vancomycin and cefepime to begin at the same time. Which antibiotic should you administer first to the patient?
   1. Vancomycin
   2. **Cefepime**
   3. It does not matter which antimicrobial is administered, so long as both antimicrobials are administered as quickly as possible
   4. Whichever medication can be infused the fastest should be administered first
   5. I should call the pharmacist or provider to clarify the correct order
5. A urine culture should **NOT** be sent for all of the following scenarios **EXCEPT**:
   1. Foul smelling or cloudy urine
   2. **Suprapubic pain and tenderness**
   3. Pre-operative screening urine culture prior to cardiac surgery
   4. Test of cure post-treatment to ensure the antibiotic we used was effective
6. Which of the following clinical scenarios would best warrant sending a urinalysis to aid in the diagnosis of a urinary tract infection (UTI)?
   1. Foul smelling or cloudy urine
   2. Altered mental status
   3. **New or worsening urinary urgency/frequency**
   4. All of the above are clinical scenarios that warrant sending a urinalysis to aid in the diagnosis of a UTI
7. Which of the following patients may warrant treatment of bacteria isolated from a urine culture in the absence of urinary symptoms (e.g., treatment of asymptomatic bacteriuria)?
   1. An elderly woman with altered mental status
   2. An elderly man with cloudy, foul smelling urine
   3. **A pregnant woman**
   4. An individual who is immunocompromised
8. True or false: patients with reported penicillin allergies should not receive cephalosporins as they will likely experience cross-reactivity with both classes of drugs.
   1. True
   2. **False**

The following statements/questions seek to understand your experience with antimicrobial educational resources and training. To what extent are you familiar with the following resources?

| **Statement** | **Very familiar** | **Familiar** | **Neutral** | **Unfamiliar** | **Very unfamiliar** |
| --- | --- | --- | --- | --- | --- |
| CustomID | 1 | 2 | 3 | 4 | 5 |
| IDSA Guidelines | 1 | 2 | 3 | 4 | 5 |
| Facility Antibiogram | 1 | 2 | 3 | 4 | 5 |
| ASET Team Pager | 1 | 2 | 3 | 4 | 5 |

IDSA: Infectious Diseases Society of America

ASET: Antimicrobial Stewardship and Evaluation Team

The following statements/questions seek to understand your experience with sources of antimicrobial expertise. How likely are you to rely on the following sources of information about the treatment of infectious diseases?

| **Statement** | **Very likely** | **Likely** | **Somewhat Likely** | **Unlikely** | **Very Unlikely** |
| --- | --- | --- | --- | --- | --- |
| Textbooks | 1 | 2 | 3 | 4 | 5 |
| ID physicians | 1 | 2 | 3 | 4 | 5 |
| Non-ID physicians | 1 | 2 | 3 | 4 | 5 |
| ID pharmacists | 1 | 2 | 3 | 4 | 5 |
| Non-ID pharmacists | 1 | 2 | 3 | 4 | 5 |
| Infection prevention | 1 | 2 | 3 | 4 | 5 |
| Colleague | 1 | 2 | 3 | 4 | 5 |
| Personal experience | 1 | 2 | 3 | 4 | 5 |
| PubMed | 1 | 2 | 3 | 4 | 5 |
| Up-to-Date | 1 | 2 | 3 | 4 | 5 |
| Internet search/Google | 1 | 2 | 3 | 4 | 5 |

Thank you for completing this survey! We sincerely appreciate your time and input. If you have any questions regarding this survey or subsequent output from this survey, please contact Jillian Hayes ([jillian.hayes@duke.edu](mailto:jillian.hayes@duke.edu)).

Supplementary Results:

**Survey Disseminated**: January 9-March 31, 2023

**Responses**: 128

- Exclusions:
  - Campus other than DUH (n=6)
  - Incomplete response (n=37)
- Complete Survey Responses: 85

**Demographics**

| Category | n (%) |
| --- | --- |
| Degree  Associates or Diploma  Bachelors  Masters or higher | 8  63  14 |
| Primary Role  Staff Nurse  Charge Nurse  Assistant Nurse Manager  Nurse Manager  Preceptor  Infection Prevention | 56  10  10  6  2  1 |
| Shift  Dayshift  Nightshift  Rotating | 59  15  11 |
| Years in Nursing (years), median (IQR) | 9 (3-17) |
| Years at DUH (years)  <5  5-10  10-19  >19 | 36  22  17  10 |
| Nursing Specialty  Surgery  Critical Care  Other*  Oncology  Medicine/Pulmonology  Cardiology  Neurology  Infection Prevention  Administration  Emergency Medicine  OB/GYN | 24  12  11  10  9  8  5  3  1  1  1 |

*Other (11): Dialysis x 2, Behavioral, Ambulatory x 3, Radiology, Vascular Access, Float Pool, Endocrinology, Cath Lab

**Attitudes and Practices: US**

| **Statement** | **Strongly Agree** | **Agree** | **Neutral** | **Disagree** | **Strongly Disagree** |
| --- | --- | --- | --- | --- | --- |
| Antimicrobial resistance is a significant problem nationally. | 32 | 46 | 6 | 1 | 0 |
| Antimicrobial use can harm patients. | 23 | 36 | 14 | 10 | 2 |
| Antimicrobials are overused nationally. | 20 | 38 | 18 | 9 | 0 |
| Selecting broad-spectrum antimicrobials when narrower spectrum antimicrobials are available contributes to antimicrobial resistance. | 26 | 42 | 14 | 3 | 0 |
| Antimicrobial resistance can be reduced by improving infection control practices. | 33 | 46 | 5 | 1 | 0 |
| Development of new antimicrobials will keep up with increasing resistance. | 11 | 32 | 22 | 17 | 3 |
| Antimicrobial stewardship programs reduce the problem of antimicrobial resistance. | 12 | 58 | 13 | 2 | 0 |
| In general, antimicrobial stewardship programs improve patient care and safety. | 25 | 48 | 11 | 1 | 0 |

**Attitudes and Practices: DUH**

| **Statement** | **Strongly Agree** | **Agree** | **Neutral** | **Disagree** | **Strongly Disagree** |
| --- | --- | --- | --- | --- | --- |
| Antimicrobial resistance is a significant problem at my institution. | 4 | 26 | 47 | 8 | 0 |
| Antimicrobials are overused at my institution. | 5 | 17 | 48 | 15 | 0 |
| Antimicrobial use can harm patients at my institution. | 11 | 41 | 24 | 9 | 0 |
| Selecting broad-spectrum antimicrobials when narrower spectrum antimicrobials are available contributes to antimicrobial resistance at my institution. | 13 | 44 | 24 | 4 | 0 |
| Antimicrobial resistance can be reduced by improving infection control practices at my institution. | 17 | 61 | 7 | 0 | 0 |
| In general, the antimicrobial stewardship program at my institution improves patient care and safety. | 21 | 50 | 14 | 0 | 0 |

**Attitudes and Practices: Personal Experiences at DUH**

| **Statement** | **Strongly Agree** | **Agree** | **Neutral** | **Disagree** | **Strongly Disagree** |
| --- | --- | --- | --- | --- | --- |
| When giving a patient an antimicrobial, I know why the patient is receiving the medication. | 28 | 48 | 8 | 1 | 0 |
| It is my responsibility to contribute to appropriate antimicrobial use at my hospital. | 25 | 49 | 9 | 1 | 1 |
| I would like more education on the appropriate use of antimicrobials. | 23 | 49 | 10 | 3 | 0 |
| Strong knowledge of antimicrobials is important in my role. | 27 | 51 | 7 | 0 | 0 |

**Confidence with Antimicrobials**

| **Statement** | **Strongly Agree** | **Agree** | **Neutral** | **Disagree** | **Strongly Disagree** |
| --- | --- | --- | --- | --- | --- |
| I am confident that I know when to recommend conversion of intravenous antibiotics to an oral formulation. | 4 | 18 | 26 | 22 | 15 |
| I am confident that I know how to perform a thorough antimicrobial allergy assessment. | 10 | 22 | 15 | 27 | 11 |
| I am confident that I know the best order to administer medications when vancomycin and a beta-lactam are ordered to start at the same time in a patient presenting with infection. | 11 | 17 | 14 | 24 | 19 |
| I am confident that I know when to obtain a urinalysis for a patient. | 21 | 38 | 9 | 9 | 8 |
| I am confident that I can categorize antimicrobials appropriately (e.g., beta-lactams, fluoroquinolones, etc.) | 3 | 17 | 15 | 29 | 21 |

**Results: Knowledge-Based Questions**

| **Question** | **Correct Responses, n (%)** |
| --- | --- |
| Which of the following antibiotics is a beta-lactam? (D) | 70 (82) |
| Which of the following antibiotics can readily be converted from IV to oral at a 1:1 dosing conversion? (B) | 47 (55) |
| Select the patient below who represents the most ideal patient to consider conversion from IV to oral antimicrobials. (B) | 82 (96) |
| You are caring for a patient presenting with sepsis from a urinary source. The primary team has ordered both vancomycin and cefepime to begin at the same time. Which antibiotic should you administer first to the patient? (B) | 30 (35) |
| A urine culture should **NOT** be sent for all of the following scenarios **EXCEPT: (B)** | 8 (9) |
| Which of the following clinical scenarios would best warrant sending a urinalysis to aid in the diagnosis of a urinary tract infection (UTI)? (C) | 3 (4) |
| Which of the following represents an appropriate way to collect a urine specimen? (B) | 31 (36) |
| Which of the following patients may warrant treatment of bacteria isolated from a urine culture in the absence of urinary symptoms (e.g., treatment of asymptomatic bacteriuria)? (C) | 13 (15) |
| True or false: patients with reported penicillin allergies should not receive cephalosporins as they will likely experience cross-reactivity with both classes of drugs. (False) | 30 (35) |

| **Statement** | **Strongly Agree** | **Agree** | **Neutral** | **Disagree** | **Strongly Disagree** |
| --- | --- | --- | --- | --- | --- |
| Confidence in Ability to Answer the Above Questions without Consultation of Resources | 4 | 11 | 22 | 35 | 13 |

**ASP Resource Familiarity**

| **Statement** | **Very familiar** | **Familiar** | **Neutral** | **Unfamiliar** | **Very unfamiliar** |
| --- | --- | --- | --- | --- | --- |
| CustomID | 0 | 5 | 6 | 33 | 41 |
| IDSA Guidelines | 0 | 2 | 9 | 35 | 39 |
| Facility Antibiogram | 0 | 3 | 9 | 30 | 43 |
| ASET Team Pager | 0 | 5 | 6 | 33 | 41 |

Likelihood to Consult Resources

| **Statement** | **Very likely** | **Likely** | **Neutral** | **Unlikely** | **Very Unlikely** |
| --- | --- | --- | --- | --- | --- |
| Textbooks | 6 | 28 | 16 | 23 | 12 |
| ID physicians | 29 | 37 | 11 | 5 | 3 |
| Non-ID physicians | 5 | 34 | 34 | 10 | 2 |
| ID pharmacists | 37 | 32 | 7 | 6 | 3 |
| Non-ID pharmacists | 8 | 40 | 29 | 6 | 2 |
| Infection prevention | 25 | 41 | 13 | 3 | 3 |
| Colleague | 12 | 36 | 28 | 9 | 0 |
| Personal experience | 4 | 30 | 37 | 14 | 0 |
| PubMed | 7 | 33 | 24 | 13 | 8 |
| Up-to-Date | 9 | 35 | 21 | 13 | 7 |
| Internet search/Google | 8 | 32 | 18 | 22 | 5 |
